# Magnetically actuated glaucoma drainage device for regulating intraocular pressure after implantation

**DOI:** 10.1038/s41378-023-00561-9

**Published:** 2023-07-20

**Authors:** Inês C. F. Pereira, Ralph J. S. van Mechelen, Hans M. Wyss, Leonard Pinchuk, Henny J. M. Beckers, Jaap M. J. den Toonder

**Affiliations:** 1 Microsystems, Department of Mechanical Engineering, Eindhoven University of Technology, 5600MB Eindhoven, The Netherlands; 2 Institute for Complex Molecular Systems (ICMS), Eindhoven University of Technology, 5600MB Eindhoven, The Netherlands; 3 University Eye Clinic Maastricht, Maastricht University Medical Centre+ (MUMC+), 6202AZ Maastricht, The Netherlands; 4 InnFocus, Inc., a Santen Company, Miami, Florida 33186 USA; 5 Ophthalmic Biophysics Center, Bascom Palmer Eye Institute, University of Miami Miller School of Medicine, Miami, Florida 33136 USA

**Keywords:** Engineering, Materials science

## Abstract

The key risk factor for glaucoma is increased intraocular pressure (IOP). Glaucoma drainage devices implanted in the eye can reduce IOP and thus stop disease progression. However, most devices currently used in clinical practice are passive and do not allow for postsurgical IOP control, which may result in serious complications such as hypotony (i.e., excessively low IOP). To enable noninvasive IOP control, we demonstrate a novel, miniature glaucoma implant that will enable the repeated adjustment of the hydrodynamic resistance after implantation. This is achieved by integrating a magnetic microvalve containing a micropencil-shaped plug that is moved using an external magnet, thereby opening or closing fluidic channels. The microplug is made from biocompatible poly(styrene-*block*-isobutylene-*block*-styrene) (SIBS) containing iron microparticles. The complete implant consists of an SIBS drainage tube and a housing element containing the microvalve and fabricated with hot embossing using femtosecond laser-machined glass molds. Using in vitro and ex vivo microfluidic experiments, we demonstrate that when the microvalve is closed, it can provide sufficient hydrodynamic resistance to overcome hypotony. Valve function is repeatable and stable over time. Due to its small size, our implant is a promising, safe, easy-to-implant, minimally invasive glaucoma surgery device.

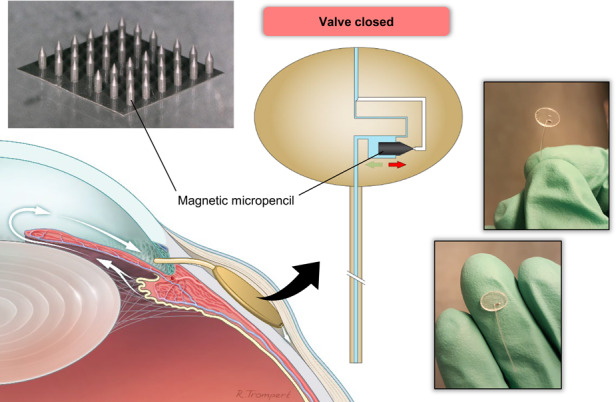

## Introduction

**Fig. 1:**
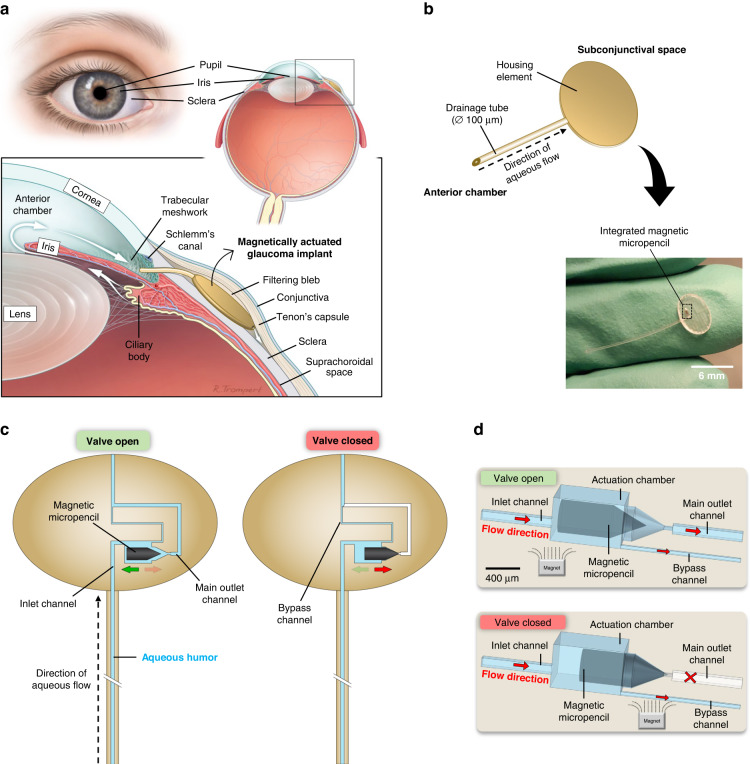
Placement of the magnetically actuated glaucoma implant in the eye and its design and working principle. **a** Anatomy of the eye and placement of the implant in the eye (image provided by Rogier Trompert Medical Art). **b** Schematic depiction of the magnetically adjustable glaucoma implant design and a photo of the actual device. **c** Front view of the implant showing a schematic representation of its channel layout and of the working principle of the integrated valving system. **d** Three-dimensional, zoomed view of the microvalve demonstrating the actuation mechanism of the magnetic micropencil; the total length and largest diameter of the micropencil are 1 mm and 357 μm, respectively

Glaucoma is a chronic and progressive eye disease characterized by damage to the optic nerve and visual field loss. With over 70 million people affected worldwide (10% eventually bilaterally blind), glaucoma is the leading cause of irreversible blindness^1,2^. This number is expected to increase to over 100 million people affected by 2040^3^. The key risk factor for the development and progression of glaucoma is elevated intraocular pressure (IOP), which results from a malfunctioning of the fluidic system of the eye designated to maintain a balanced amount of aqueous humor in the anterior chamber^4–6^. The anatomy of the eye is schematically depicted in Fig. 1a. The aqueous humor is produced and secreted by the ciliary body and mainly drains out of the eye through the trabecular meshwork and into Schlemm’s canal. In patients with primary open-angle glaucoma (POAG), there is an abnormal increase in resistance to aqueous outflow, which leads to a buildup of fluid in the eye that results in elevated IOP. An IOP >21 mmHg is generally considered abnormally high. Recent clinical trials have designated “success” as IOP levels <18, <15, or even <12 mmHg^7^. In this paper, we consider 15 mmHg as the upper limit of the acceptable IOP range. Currently, IOP is the only risk factor that can be modified to stop the progression of glaucoma. Ophthalmologists use a variety of approaches to decrease IOP, including pharmacological medication, laser procedures, and/or incisional surgeries^8^. Surgery is often performed when the maximum tolerated medical/laser treatments do not sufficiently reduce IOP^9^. The standard surgical paradigm involves bypassing the eye’s natural outflow pathways by creating an alternative route for the aqueous humor to effectively exit the eye, thereby reducing IOP. Conventional filtration surgeries include trabeculectomy and the implantation of glaucoma drainage devices. Most glaucoma implants, including conventional aqueous shunts (with tube-plate design) and some of the new less invasive bleb-forming devices, drain the aqueous humor into the subconjunctival/sub-Tenon’s space, where a fluid reservoir known as a filtering bleb is formed.

Although better-designed glaucoma implants have emerged in recent years and are made of superior materials that evoke less tissue inflammation, postoperative complications following glaucoma filtration surgery are still frequently reported; the two most common complications are excessive fibrosis with scar tissue formation and hypotony-related complications^1,10–14^. Hypotony, commonly defined as an IOP lower than 5 mmHg, is a known complication that is associated with glaucoma filtration procedures and most often occurs in the initial postoperative phase. Common causes include conjunctival bleb leak and overfiltration of aqueous humor through the implanted device due to the absence of a “maturated” filtering bleb in the early postoperative period^15^. During bleb maturation, the balanced production and degradation of the extracellular matrix and collagen produce a tissue-strengthening effect, which limits the filtration of aqueous humor from the bleb into the subconjunctival tissue^16^. Until this phase is reached, which might take up to a few months, there may be overfiltration of aqueous humor, resulting in hypotony^17^. This vision-threatening condition may lead to vision loss in up to 20% of patients, and it can be accompanied by a shallow anterior chamber and other complications, such as choroidal effusions and suprachoroidal hemorrhage^18^. Many flow restricting techniques have been extensively used by ophthalmologists to prevent overdrainage in the early period after surgery, and these include, for instance, placing an intraluminal stent inside the implant’s tube and then removing it postoperatively a few months after surgery or ligating the tube externally with an absorbable ligature that degrades after ~6 weeks. This procedure significantly reduces flow, thus enabling the formation of a tissue capsule over the plate of the implant, which then offers some resistance to aqueous humor outflow^19^. A main drawback to these measures, however, is the lack of precision and predictability in efficiently controlling IOP in the initial postoperative phase, as shown by the regular occurrence of early hypotony^10,20,21^.

To overcome this lack of postoperative IOP control, some attempts have been made to incorporate valves into glaucoma implants. An example of a valved implant is the Ahmed® Glaucoma Valve (New World Medical, Inc., California, USA), a marketed device that features a built-in Venturi-based passive valve^22,23^. Although complications associated with overfiltration and subsequent hypotony appear to occur less frequently with this device, evidence suggests that hypotony continues to affect a significant proportion of patients^10,13,14,24^. Another example of a valved glaucoma implant that has recently been made commercially available, is the eyeWatch™ Implant (Rheon Medical SA, Lausanne, Switzerland), which incorporates an active magnetic valve. The eyeWatch system features the eyeWatch implant, which acts as an adjustable faucet that controls IOP, and the eyeWatch Pen, which is used to tune the flow resistance of the implant by inducing variable compression of the drainage tube. This compression is achieved by rotating a magnetic disk present inside the implant^25,26^. Initial clinical results with eyeWatch suggest that it can prevent hypotony and hypotony-related complications from occurring, and IOP spikes are avoided by fine-tuning the flow resistance of the device when needed, thus promoting smooth pressure transitions that may mitigate the tissue response^18,27^. Nonetheless, further studies involving a significant number of patients and longer follow-up times are necessary to support the long-term safety, efficacy, and clinical relevance of this device in comparison with other implants.

In this work, we have developed a novel miniature magnetically controlled glaucoma drainage device where, unlike the eyeWatch system that requires compression/decompression of a tube to adjust fluidic resistance, we rely on a pencil-tip shaped actuator that selectively opens a larger fluid passageway in the device to lower IOP. A potential advantage of our device is the small size of the integrated magnetic microvalve, which enables a smaller overall implant size. Our implant is made from poly(styrene-*block*-isobutylene-*block*-styrene) or ‘SIBS’, which is highly biocompatible due to its inertness, softness and flexibility. It is the thermoplastic elastomer comprising the PRESERFLO® MicroShunt (Santen, Osaka, Japan) implant, which has been used in humans since 2007 and demonstrates, for the most part, less inflammation and scar tissue formation^28–31^. The movable magnetic microplug that is part of our valving system is also fabricated using SIBS mixed with magnetic microparticles. Being only partly composed of magnetic material, and due to its small dimensions, the magnetic micropencil plug is very light, and as a result, the overall implant might also be lighter and more flexible than other currently available implants. Our approach provides a promising route toward a miniature implant that will enable the ophthalmologist to precisely and actively adjust the IOP to a desired, healthy range. The outflow of aqueous humor can be kept to a minimum in the immediate postoperative period until the ophthalmologist determines that the bleb is sufficiently mature, thus preventing hypotony, and can be increased to a maximum afterward to further lower the IOP and stop glaucoma disease progression.

### Design and working principle of the magnetically actuated glaucoma implant

Our active, magnetically adjustable glaucoma drainage device comprises a drainage tube and a housing element, as shown in Fig. 1b. The housing element is attached to the outlet of the drainage tube and contains an actuation chamber where the magnetic plug with the shape of a “micropencil” is integrated. Figure 1a shows the implantation site of the glaucoma implant, where the entrance to the drainage tube is in the anterior chamber and the plate is implanted under the conjunctiva and Tenon’s capsule. Aqueous humor flows through the tube and into the plate through the magnetically actuated valve system and then out of the plate to the surrounding tissue. The details of the magnetically actuated valve system are shown in Fig. 1c, d. The system is implanted with the valve closed to minimize the chance of hypotony. In this closed “low-flow, high hydrodynamic resistance” configuration, which is demonstrated in Fig. 1c – right and Fig. 1d – bottom, aqueous humor flows through the tube, into the inlet channel, and then to the bypass channel and out of the plate to fill the bleb. Once the bleb is matured, the surgeon can actuate the valve system with an external magnet and move the magnetically coupled pencil-shaped plug to the left, as shown in Fig. 1c – left and Fig. 1d – top, thereby opening the “high-flow, low hydrodynamic resistance” main outlet channel to increase flow and decrease IOP. Note that the bypass channel remains open in both low-flow and high-flow modes and has a cross-sectional area smaller than the main outlet channel. The dimensions of this channel were predetermined to achieve a desired minimum pressure in the case of hypotony when the device was in the low-flow mode. According to our previously described numerical model^17^, to obtain a healthy IOP of 10 mmHg, the bypass channel must have a cross-sectional area of 50 × 50 µm (width × height). However, this optimal pressure can only be attained if the micropencil totally stops the flow through the main outlet channel. As we anticipated that some fluid leakage might occur around the micropencil in the closed state, we fabricated two types of devices with different bypass channel dimensions: 50 × 50 µm and 40 × 40 µm. The inlet channel and main outlet channel were designed with a cross-sectional area of 100 × 100 µm. The actuation chamber in which the micropencil is integrated comprises a rectangular portion that opens to a channel with the same round tapered shape as the tip of the micropencil. As shown in Fig. 1c, d, approximately 200 µm of the micropencil tip is already positioned inside the micropencil channel of the actuation chamber when the valve is in the open state. This was intentionally designed this way to ensure that the micropencil is always properly aligned with its direction of movement.

## Results

### Micropencil plug and in vitro device fabrication

**Fig. 2:**
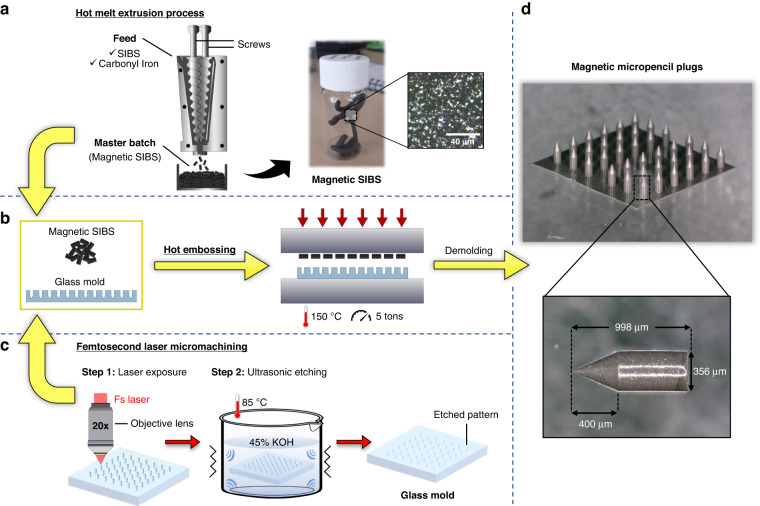
Fabrication process of the magnetic micropencil plug and its final shape and dimensions. **a** Illustration of the hot melt extrusion process used to fabricate the magnetic SIBS pellets, which show a relatively uniform dispersion of the magnetic particles in the SIBS matrix, as seen in the microscopy image on the right. **b** Schematic representation of the micropencil fabrication by replica molding using hot embossing with femtosecond laser-machined fused silica glass molds. **c** Schematic illustration of the femtosecond laser machining process used to fabricate the glass molds. **d** Demolded array of magnetic micropencils and shape and dimensions of the micropencil plug

The magnetic micropencil plugs were fabricated by replica molding using hot embossing, as illustrated in Fig. 2b. The micropencils were made of magnetic SIBS, a composite material comprised of SIBS mixed with carbonyl iron powder and fabricated upfront using a hot melt extrusion process, as shown in Fig. 2a. By employing proper mixing parameters, we were able to achieve a good dispersion of the magnetic particles in the SIBS matrix (see Fig. S1 for microscopic images showing the homogeneity of the particle distribution in SIBS, taken from a 100-µm-thick hot embossed magnetic SIBS film and captured using varying magnifications in both transmission and reflection modes for imaging the samples). An experiment was performed to investigate the cytotoxicity of the magnetic SIBS, which confirmed that it is noncytotoxic (see the Supplementary Information for more details about this experiment and Fig. S2 for the corresponding results). The mold used to give the magnetic SIBS a micropencil shape was fabricated by femtosecond laser micromachining of fused silica glass. Femtosecond laser-assisted chemical wet etching is based on a two-step process of ultrashort-pulsed laser radiation in transparent materials, followed by chemical wet etching to selectively remove the exposed material (Fig. 2c)^32^. The laser beam, focused inside the glass, locally modifies its refractive index and chemical properties and creates patterns that can be used to generate three-dimensional structures with high precision, aspect ratio, and complexity by chemical etching^32,33^. The complexity of the pencil shape of our microplugs would be difficult to achieve using other classical micromanufacturing techniques, such as photolithography. The glass mold was designed to produce micropencils with a total length of 1 mm, a 400-µm-long conical tip and a diameter of 350 µm. As shown in Fig. 2d, the fabricated micropencils have tolerances for length and diameter of 356 ± 1 µm and 988.3 ± 10 µm, respectively. These discrepancies between the designed and measured dimensions are due to the nature of the femtosecond laser machining process, as explained in more detail in the Supplementary Information. Our experiments indicate that these tolerances do not appear to affect the valve function, provided that the length of the plugs is sufficient to maintain their tips partially inside the micropencil opening of the actuation chamber when the valve is in the open state, thus keeping them aligned with the movement direction.

**Fig. 3:**
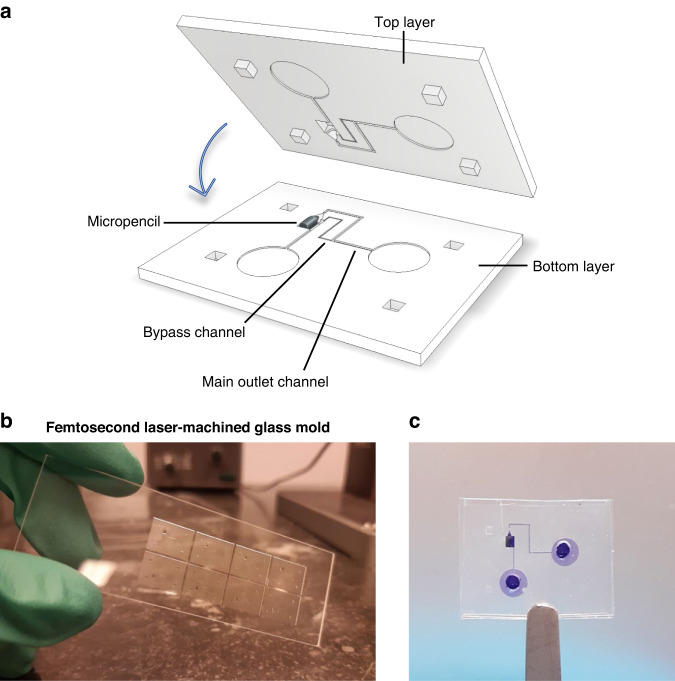
Micropencil microfluidic device used for the in vitro experiments. **a** An exploded view of the micropencil device with an integrated valving system, showing the three components being the top layer, the magnetic micropencil, and the bottom layer. The cross-sectional areas of the main outlet channel and bypass channel are 100 × 100 µm and 50 × 50 or 40 × 40 µm, respectively. **b** Picture of the glass mold, made using femtosecond laser machining, used for the fabrication of top and bottom layers. **c** Picture of the bonded micropencil device filled with ink

To investigate the actuation of the micropencil in vitro, we fabricated a microfluidic device with the same channel design and dimensions that will be present in the final implant’s housing element (shown in Fig. 1c and later in Fig. 5a). Figure 3a illustrates the three components – top layer, magnetic micropencil and bottom layer – that comprise the device and how they are assembled to obtain a closed device (also shown in Supplementary Fig. S3a, b). Both layers contain half of the main outlet and bypass channels and the actuation chamber in which the micropencil is positioned, thereby establishing the microvalve. They were fabricated by replica molding using hot embossing and a femtosecond laser-machined fused silica glass mold (Fig. 3b), following the same procedure used for fabricating the micropencil plugs. Figure 3c shows a picture of the bonded micropencil device filled with ink, identifying the channels and the actuation chamber. The design and dimensions of the microfluidic device and respective channels are represented in Supplementary Fig. S3c, d. The height and width of both the main outlet and bypass channels were measured, and the results can be found in Supplementary Fig. S3e. The measured heights of the channels are very similar to the designed values, whereas the measured widths are slightly smaller than the designed widths. Again, these differences are due to the nature of the femtosecond laser machining process (see the Supplementary Information).

### In vitro performance of the micropencil device

**Fig. 4:**
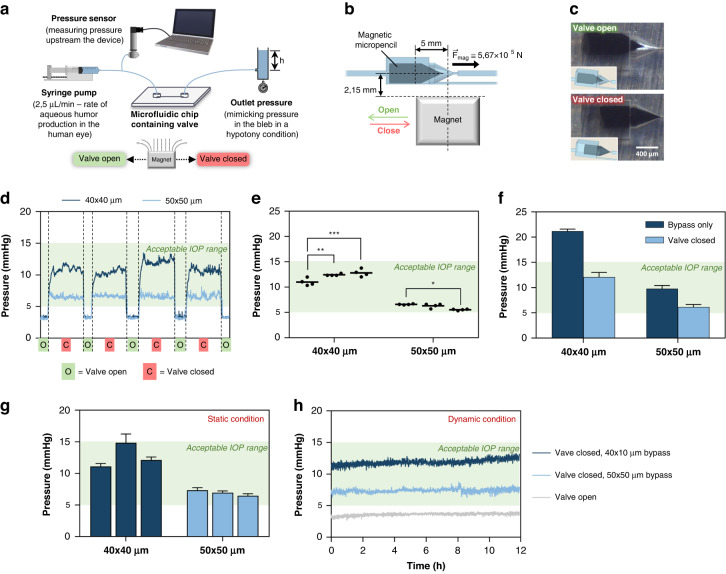
In vitro microfluidic experiments. **a** Schematic depiction of the setup used for the microfluidic experiments. **b** Magnet placement relative to the micropencil plug when the plug is to be moved to the closed state (${\vec{\text{F}}}_{\text{mag}}$ represents the magnetic translational force applied to the plug, calculated as described in the Supplementary Information). **c** Microscopic images showing the microvalve in the open and closed states. **d** In vitro measurement of the pressure variation upstream of the 40 × 40 µm and 50 × 50 µm micropencil devices as a result of valve operation. In this experiment, the pressure was measured for ~5 min with the valve open and 30 min with the valve closed. **e** Overview of the pressures measured in each valve-closed cycle for all individual samples tested. Each data point represents the pressure measured in each valve-closed cycle, and the straight line represents the mean (*n* = 4 cycles per sample). *, **, and *** represent *p* ≤ 0.05, *p* ≤ 0.01, and *p* ≤ 0.001, respectively, as analyzed by two-way ANOVA with Bonferroni multiple compariso*n*s test (*n* = 3 samples per group). **f** Comparison between the pressures measured in the devices with the valve in the closed state and the devices containing the bypass channel only, i.e., without the main outlet channel and the integrated microvalve, representing a perfectly closed valve. The data represent the mean ± standard deviation (SD) (n = 3). **g** Overview of the pressures measured upstream of the 40 × 40 µm and 50 × 50 µm micropencil devices for a period of 12 h with the valve in the closed state under static conditions (i.e., the devices did not move over the course of the experiment). Each column represents one sample, and the error bars indicate the variation in the pressure throughout the experiment. **h** Pressure measured upstream of the 40 × 40 µm and 50 × 50 µm micropencil devices placed under rocking motion (dynamic condition) for a period of 12 h with the valve in the closed and open states. In all graphs, the shaded green areas represent an acceptable IOP range of 5–15 mmHg

The micropencil devices were evaluated in vitro to determine if the valve function is repeatable and stable over time and if the devices provide stable pressures under nonstatic conditions in either open or closed states. The setup used to carry out these experiments is schematically represented in Fig. 4a. Deionized (DI) water was pumped into the devices at 2.5 µL/min, the rate of aqueous humor production in the human eye^34^, and the inlet pressure, which would correspond to the IOP, was measured in real time. The value of 2.5 µL/min was chosen as an approximate average value of the aqueous humor production rate during a period of 24 h^35^. As our glaucoma implant was designed to prevent hypotony from occurring, we simulated a hypotony situation in our in vitro experiments to test the performance of our device and integrated microvalve in a real hypotony case scenario. To simulate this condition, the outlet of the devices was connected to a column of water mimicking the pressure in the bleb when hypotony is most likely to occur (~1.38 mmHg, or 1.88 cmH_2_O). This pressure was calculated in our previous work^17^.

First, to evaluate the repeatability of the valving system, we performed a cycle switching test wherein the microvalve was switched between open and closed states four times by moving the micropencil plug using an external magnet while continuously measuring the pressure upstream from the device. The magnet was placed on either side of the micropencil device, and the plug was moved to the open or closed positions accordingly (the magnet was not dragged between these two locations). A schematic depiction of the position of the magnet relative to the micropencil plug, particularly when the plug is to be moved to the closed state, is shown in Fig. 4b. The magnetic translational force acting on the plug at this position, i.e., at a distance of 5 mm between the center axis of the magnet and the plug, is ~5.67 × 10^−5^ N (the calculation of the magnetic force is explained in detail in the Supplementary Information). This force is a combined effect of the magnitude of the magnetic field and the magnetic field gradient experienced by the plug, which, at a distance of 5 mm, are $\text{B}$ = 0.27 T and *∇* $\text{B}$ = 88.77 T/m, respectively. The calculated magnetic translational force is the resultant force necessary to move the plug when not considering static and kinetic friction acting on the plug due to the sticky nature of SIBS. Since this stickiness and its effect on the plug’s movement is significantly reduced by the constant presence of the liquid environment surrounding the plug, we did not consider these forces in our calculations. Indeed, if the device is completely dry, moving the microplug seems impossible due to the major role of the friction forces in that scenario. However, once fluid is injected into the device, the plug begins to move almost immediately under the influence of the applied magnetic field.

Since the placement of the magnet was done manually, we could never precisely place it at a distance of 5 mm from the plug where the maximum magnetic translational force is achieved. Nevertheless, the valve always moved as intended, which confirms that the valving can be controlled robustly, and the exact positioning is not critical. In a final product, however, we conceive that the magnet is integrated in a controller device that is placed on the eye and in which the magnet is easily and robustly translated between two positions (corresponding to open and closed valve positions). Such a controller device could additionally detect the shape of the end-plate of the implant under the conjunctiva and precisely position the magnet on either side of the major axis of the ellipse-shaped end-plate at a distance of 5 mm from the plug.

Figure 4c shows microscopic images of the microvalve in the open and closed states, and the movement of the micropencil between these two states is shown in Movie S1. Two different types of devices were tested in this cycle switching test: one containing the 50 × 50 µm bypass channel and the other with the 40 × 40 µm bypass channel. For both devices, measurements were performed on three different samples. The pressure results of one of the 50 × 50 µm and 40 × 40 µm devices tested are presented in Fig. 4d, and the results for the other devices can be found in Supplementary Fig. S4. From these measurements, we can confirm that both types of devices are able to increase the pressure when the valve is switched to the closed state, from the very low pressure characteristic from a hypotony situation ( < 5 mmHg) to a pressure within the acceptable IOP range (5–15 mmHg, shaded green area in the graph of Fig. 4d). The very low pressure measured when the device is fully open, ~3.6 mmHg, indicates that the device should be implanted in the eye with the valve in the closed state to effectively prevent hypotony from occurring in the first postoperative weeks and then switched to the open state when the doctor determines, by measuring the IOP in the follow-up visits, that the bleb is sufficiently mature to offer enough resistance to the outflow of aqueous humor. Figure 4e shows an overview of the pressures measured in each valve-closed cycle for all individual samples tested. These results show that the 50 × 50 µm micropencil device provides pressures in the range of 5–10 mmHg when the valve is closed, whereas the 40 × 40 µm device offers higher pressures in the range of 10–15 mmHg. Both devices showed good repeatability, both between cycles and between different samples. This indicates that the different magnetic SIBS valves that we tested always behaved identically, which is a clear indication that the magnetic particles are homogeneously dispersed within the SIBS polymer. Otherwise, a poorly mixed composite could have negatively affected actuation performance and led to variations between pressure measurements. When comparing the 50 × 50 µm and the 40 × 40 µm devices, the former showed the best repeatability. The pressure varied slightly more between measurements in the 40 × 40 µm device, which may be explained by a possible deformation of channels as a result of the higher fluidic pressures inside this device when the valve is closed. Due to the flexible nature of SIBS, these higher pressures might lead to a slight expansion of the actuation chamber, which can result in a less stable closure of the valve. In addition, the proper function of the valve is highly dependent upon the correct fitting of the micropencil into the micropencil opening of the actuation chamber. A slight mispositioning could result in different outflow resistances and therefore different valve-closed pressures. This alignment might also explain the slight differences between pressures measured in the closed state for all devices tested. However, these differences are not significant and are acceptable for the final application as a glaucoma drainage device.

To investigate whether fluid can still leak past the micropencil and through the main outlet channel when the valve is closed, we fabricated and tested devices without the main outlet channel and without the microvalve integrated. The pressure measured with these devices should correspond to the pressure that our microvalve would provide if it would perfectly close and completely stop the flow through the main outlet channel. This pressure was then compared with that obtained in the closed-state device. The results are plotted in Fig. 4f. Our findings show that fluid can still flow through the microvalve, past the micropencil and into the main outlet channel when the valve is in the closed state, thus indicating that the valve is leaky. To avoid leakage, the mechanical properties of the plug could be adjusted; for instance, a more elastic material could (possibly) create a better seal with less leakage. Increasing the elasticity (i.e., lowering the elastic modulus) of the composite could be achieved in two ways: (1) decreasing the amount of magnetic particles in the SIBS matrix, or (2) decreasing the styrene content of the SIBS starting material (which, with 25% styrene content as used in this work, has a Young’s modulus of 10 MPa – value provided by InnFocus Inc.). The first option may negatively affect the valve actuation since the magnetic susceptibility of the composite is reduced; this means that the composite would be less responsive to an external magnetic field. The second option, i.e., a lower content of styrene, could lead to an increase in adhesion between the plug and the valve chamber, which could also negatively affect actuation. The optimization of the mechanical properties is therefore a delicate balance between various factors and could be a future optimization of the valve.

We theoretically determined the hydrodynamic resistance of both the 40 × 40 and 50 × 50 µm devices with the valve in the open and closed states. The hydrodynamic resistance provided by the valve in both devices was also calculated, and the obtained values are almost identical, confirming that the functioning of the valve does not depend on the bypass channel dimensions. A detailed description of the equations used for these calculations and corresponding results are provided in the Supplementary Information and Table S1.

To evaluate the stability of the pressure when the valve is closed, we measured the pressure upstream of the two types of devices for a period of 12 h. Figure 4g shows the results of this experiment, where each column represents one sample, and the error bars indicate the variation in the pressure throughout the experiment. The measured pressures plotted over time can be found in Supplementary Fig. S5. Except for one device, the pressure was kept remarkably stable over time, as indicated by the minor size of the error bars. This demonstrates that our micropencil is capable of maintaining a steady position in the closed state, thus keeping the pressure barely unchanged over the course of the experiment. The greatest pressure variation (~2 mmHg) in one of the 40 × 40-µm devices could have been due to a small air bubble that entered into our setup during the experiment and ended up inside the device. Such a trapped bubble will act as an additional fluidic resistance by reducing the diameter of the channels. Apart from the air bubble, an impurity flowing into the device and partially clogging its channels could have also been the reason for this pressure fluctuation. It has been reported in the literature that when implanting glaucoma drainage devices, the lumen of the tubed devices can be obstructed by different particulate matter, including sloughed endothelial cells, pigment from the iris, fibrin from blood clots and lens particulates. Of the particulates mentioned, endothelial cells have the largest diameter of ~40–50 µm^36^. This can be an issue, particularly for the micropencil device with the larger 50 × 50 µm bypass channel. When the valve is in the closed state, the endothelial cells transported by the aqueous humor might be able to move into the bypass channel, but they may not be capable of flowing through it and become trapped due to the comparable dimensions of the bypass and the cell, blocking the channel. If the bypass channel is small enough, such as 40 × 40 µm, the cell will probably not be able to move into it, thus becoming trapped in the actuation chamber until the valve is opened again. When the valve is open, the cell will flow through the main outlet channel since it has lower hydrodynamic resistance. Here, clogging is very unlikely to occur due to the larger dimensions of this channel.

The aforementioned experiment was performed under static conditions, i.e., the device did not move throughout the experiment. To investigate whether valve function would be affected by the patient’s movements, we repeated the previous experiment but with the devices placed under rocking motion using a digital platform rocker shaker. The rocking, side-to-side (2D) motion, was performed in the main direction of the micropencil movement at a tilt of 15° and a speed of 8 rpm, which corresponds to 16 side-to-side movements per minute. Three devices were tested: a 50 × 50-µm device with the valve in the open state and 50 × 50-µm and 40 × 40-µm devices with the valve in the closed state. The results are represented in Fig. 4h. Our data confirm that the valving system is stable under dynamic conditions in both open and closed states, as indicated by the stable pressure measured over time. This pressure stability suggests that our micropencil will not move to the open or closed states just by, for instance, the effect of gravitational forces. This stability is partially achieved due to the sticky nature of SIBS, which makes it very difficult to move the micropencil without a stronger external force being applied to induce its movement, in this case, magnetic force. Only when using an external magnet placed close enough to the device is the micropencil able to switch between closed and open states. Another aspect enhancing the stability of the valve, specifically in the closed state, is the special design of the actuation chamber itself. The “pencil” shape of the outlet portion of the actuation chamber is very similar to that of the micropencil plug, with very close dimensions in terms of the diameter and length of the conical tip. This effectively helps to hold the closed micropencil in place.

### Valve operation ex vivo

**Fig. 5:**
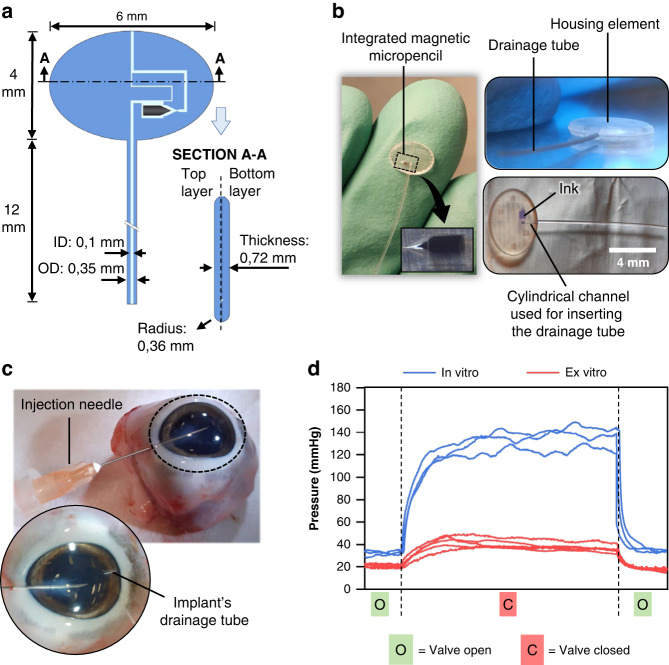
Ex vivo experiments. **a** Schematic of the magnetically actuated glaucoma implant with dimensions indicated in mm, showing a front view and a cross-sectional view. **b** Pictures of the fabricated magnetically actuated glaucoma implant with an integrated valve containing the micropencil. **c** Experimental setup used for the ex vivo experiments. **d** Red-colored lines – pressures measured in the anterior chamber of the porcine eyes as a result of the valve operation. Each dataset represents one device tested in one enucleated porcine eye; Blue-colored lines – pressures measured in vitro in the fabricated implants when applying the same flow rate used for the ex vivo experiment. Each dataset represents one device tested. In this experiment, the pressure was measured for ~10 min with the valve open and 1 h with the valve closed

We performed ex vivo experiments to better understand the performance of our glaucoma device implanted in enucleated porcine eyes. For this experiment, an actual implant comprised of a drainage tube and an elliptical-shaped end plate (or housing element) was fabricated. Figure 5a schematically depicts the design of the implant, including its dimensions indicated in mm. Figure 5b – left shows a picture of the actual magnetically actuated glaucoma device relative to a gloved finger. Figure 5b – bottom right shows the area of the plate where a channel was made to accommodate the drainage tube. With the exception of this connecting channel, all other channels in the end plate have identical designs and dimensions as in the microfluidic devices tested above. The main outlet channel extends to the edge of the plate, where it opens to the exterior. The size of the bypass channel chosen for this experiment was 40 × 40 µm.

Prior to implantation, the devices were manually injected with a saline solution by using a simple syringe to eliminate any air bubbles that may be trapped inside the channels. As a result of the fluidic pressure, accumulated bubbles will flow out of the device, and even if this process is not immediate, they gradually decrease in size until they eventually disappear. Thereafter, the devices were implanted in the eyes, which were injected with a saline solution at a constant flow rate of 20 µL/min (this approach was used by Villamarin and coworkers to test the ex vivo performance of the eyeWatch device). This specific flow rate was necessary to maintain the physiological ocular rigidity throughout the experiment and keep the anterior chamber inflated, which would otherwise lose its shape at lower flow rates due to the fluid leakage that happens all around the insertion point of the injection needle (through which fluid flows into the eye) and at the back of the enucleated eye. A cannulated eye with the implanted device is shown in Fig. 5c. This 20 µL/min flow rate resulted in an IOP of 21.12 ± 1.42 mmHg when the implant was in the high-flow mode, i.e., the valve was in the open state. After closing the valve, the IOP was raised to 39.12 ± 3.60 mmHg, as seen in the red-colored datasets of Fig. 5d. Each red-colored dataset represents the IOP measured in one eye, and each eye was implanted with a different device, meaning that in total, five eyes and five implants were used for this experiment. Overall, the results confirm once again the efficacy of our magnetically actuated implant in tuning the IOP as a result of valve operation. The very similar pressures measured in each ON and OFF cycle for the different eyes/devices tested further support our conclusions from the previous experiments, proving once more the high reproducibility of our micropencil valving system. Furthermore, compared with the ex vivo experiments performed on the eyeWatch implant^25^, the pressure difference achieved between open and closed cycles with our device of 18 mmHg is very similar to the 19.3 mmHg pressure difference provided by the eyeWatch between its fully open and fully closed configurations.

For comparison, in vitro experiments were also performed on the fabricated implants, where the same flow rate was applied as in the ex vivo experiments (20 µL/min). The results are shown in blue in the graph of Fig. 5d, where each dataset represents one device tested. In the open state, the pressure measured upstream of the devices was 32.4 ± 2.01 mmHg, which increased to 133.53 ± 8.12 mmHg when the valve was closed. The large differences observed between pressures measured in the in vitro and ex vivo experiments (Fig. 5d, blue- and red-colored lines, respectively) are to be expected, considering that, as mentioned above, in the ex vivo experiment fluid leaks all around the insertion point of the needle and at the back of the enucleated eye, thus resulting in lower IOPs. This leakage is aggravated when closing the valve: the higher the hydrodynamic resistance of the implant is, the greater the fluid leakage around the insertion point of the needle. Leakage at the back of the eye is also likely to increase as a result of the high fluidic pressures that the eye is subjected to when the valve is closed. This leakage is an unavoidable artifact of the ex vivo experiment and should not occur in vivo. One potential solution to this leakage problem is to connect a liquid reservoir to the cannula into the porcine anterior chamber instead of using a syringe pump, as we did in our experiment. By adjusting the reservoir height, different intraocular pressures could be achieved, and to investigate valve actuation, the flow rate differences at the outlet of the device could be measured. However, this experiment requires a different setup from what we currently have since we would need to apply pressure and measure flow rate instead of applying a constant flow rate and measuring pressure differences.

## Discussion

We propose herein a novel, miniature, magnetically adjustable glaucoma implant for noninvasively regulating IOP after implantation. Our implant comprises a drainage tube and a housing plate in which a microvalve, consisting of an actuation chamber containing a moveable magnetic plug with the shape of a micropencil, is integrated. By using an external magnetic stimulus, the micropencil can be moved, the microvalve is switched between two states – open and closed – and the hydrodynamic resistance of the implant is changed accordingly. The device is designed to be implanted in the eye with the valve in the closed state to provide a high hydrodynamic resistance to prevent hypotony. When the ophthalmologist determines that the filtering bleb is sufficiently mature to offer resistance to the aqueous outflow and a lower IOP is desired, the valve can be noninvasively opened with an external magnet to increase flow and decrease IOP.

Herein, we introduced a new microfabrication technique for the manufacture of this device and the integrated micropencil based upon replica molding using hot embossing and femtosecond laser-machined fused silica glass molds. The femtosecond laser machining process has proven to be an effective method to fabricate molds for both micropencil plugs and glaucoma devices. The high geometrical complexity of the micropencil shape and differing channel heights were successfully achieved using this technique. Precise machining of this nature would be extremely difficult to accomplish using other conventional micromanufacturing techniques, such as photolithography or micromilling^37^. The features in the glass molds were successfully transferred via hot embossing to the thermoplastic material SIBS, which comprises the implantable device. Hence, in this study, we have demonstrated that the combination of hot embossing of thermoplastic materials with femtosecond laser-machined glass molds is a potentially advantageous process for the mass production of microdevices that contain three-dimensional structures and require a resolution of a few micrometers, high accuracy and complexity^38^.

To investigate the performance and function of our micropencil valving system, we first performed in vitro microfluidic experiments. The results from the cycle switching tests indicated that the pressure difference achieved between open and closed states is sufficient to prevent hypotony and to maintain the IOP within an acceptable range of 5–15 mmHg. We have also confirmed that the valve function is repeatable, both within and between the samples tested herein. Further experiments demonstrated that our valving system provides stable pressures over a period of 12 h in the open and closed states under both static and dynamic conditions; the latter was achieved by placing the devices under rocking motion to simulate a patient moving. The valve stability can be explained by two factors: (1) the sticky nature of the SIBS material, which makes it very difficult to move the micropencil without an external magnetic force being applied; and (2) the design of the actuation chamber itself. The similarity and close dimensions between the “pencil” shape of the outlet portion of the actuation chamber and the tip of the micropencil plug efficiently help to hold the micropencil in place when in the closed state. Ex vivo experiments performed on enucleated porcine eyes further confirmed the efficacy and reproducibility of our magnetically actuated implant in changing the IOP by valve operation.

In future work, additional in vitro long-term measurements of the pressure over several days or months should be performed to further confirm valve stability over time. Although we have proven that our microvalve is stable under rocking motion with 16 side-to-side movements per minute at 15°, it would also be useful to investigate whether the stability of the valve is maintained under more vigorous agitation, which would better mimic, for instance, a more energetic shaking/nodding of the head, rubbing the eyes, or even accidents and body impacts. For this, the experiment performed herein could be repeated using a higher frequency of side-to-side movements and an increased angle; furthermore, to achieve these experimental conditions, a different and more powerful shaker device must be used. Moreover, the performance of our microplug may be affected by the application of compressive forces, which could occur if the implant is grasped too tightly with tweezers during the implantation procedure, for instance. To mimic these compressive forces in vitro, the device could be placed in a hot embossing machine and compressed using a limited amount of force and thereafter tested to investigate whether the actuation of the microvalve and the overall performance of the device are preserved. Furthermore, as mentioned earlier, occlusion with cells and other particulate matter might also be an issue for this type of small-lumen device, and therefore, more studies are needed to determine whether our implant is at risk of blockage. This potential clogging issue could be explored in vitro by injecting fluorescent microparticles into the device and observing if they easily flow out of it or accumulate in the channels. Long-term clogging could also arise from the accumulation of proteins present in the aqueous humor in the small channels of our device. To investigate proteinaceous biofouling over time, an in vitro experiment could be performed where a proteinaceous liquid mimicking the aqueous humor, such as fluorescent-tagged bovine serum albumin, is injected into the device. The formation of a proteinaceous film on the inner surface of the channels can then be determined using fluorescence microscopy, as demonstrated by Park et al.^39^. To reduce the risk of blockage, the microchannel design can be further adjusted to feature smoother and curved corners rather than sharp corners, which are more prone to accumulate debris. Moreover, as our device is still in the proof-of-principle stage of development, we did not include a curved end-plate to accommodate the global curvature. However, if future in vivo experiments indicate the importance of this feature, our powerful femtosecond laser machining technique can easily modify the glass mold to create the desired curvature in the end plate. The end-plate edges were already designed and fabricated with a rounded shape, as depicted in Fig. 5a, b, to avoid the potential fibrotic response caused by sharp edges. This demonstrates our capability to create intricate features in our glass mold, which can be accurately replicated in our devices.

To summarize, in the future, we plan to test our implant in an experimental animal model to confirm the laboratory results and to further evaluate the biocompatibility, controllability, and efficacy of our device. These studies would further validate the stability of the valving mechanism when placed in a living eye. Our ultimate goal is to conduct clinical trials in glaucomatous patients with uncontrolled IOPs requiring a filtering procedure. In a first clinical trial, the MRI compatibility of our device should also be investigated. Patients receiving our device should undergo an MRI scan, as performed by Roy and coworkers in the first clinical study performed on the eyeWatch implant^27^. The results from this study indicated that patients with the eyeWatch implanted experienced no discomfort or pain during MRI, with only minor image artifacts observed. However, the valve’s positioning required adjustment following the scan. We anticipate that a similar adjustment may be necessary for our device. Therefore, patients should schedule a follow-up visit with their doctor after undergoing an MRI scan to ensure proper valve positioning, if needed.

In conclusion, we have successfully developed a new molding technique that enabled the fabrication of a magnetically actuated glaucoma implant capable of switching between two hydrodynamic resistances to prevent hypotony from occurring in the early period after surgery and, if warranted, to allow for maximum outflow capacity at a later stage to effectively reduce IOP and stop glaucoma disease progression. By switching between hydrodynamic resistances in a noninvasive and nontraumatic way, we expect that our magnetically actuated glaucoma implant will be suitable for patients with mild-to-severe glaucoma, ranging from primary open angle glaucoma, where large drops in IOP are needed, to normal tension glaucoma, where fine control of relatively low IOP is required^40^. The new valving system proposed in this study can also be used for other microfluidic applications, such as in lab-on-chip and organ-on-chip systems and in controlled drug delivery devices, among others.

## Materials and methods

### Micropencil plug fabrication

The magnetic micropencil plugs were fabricated from SIBS containing homogeneously dispersed magnetic microparticles and carbonyl iron powder (CIP, 99.5%, average diameter of 5 µm, Sigma‒Aldrich). The weight ratio between SIBS and CIP was 1:2, and it was predetermined following the critical particle volume concentration (CPVC) principle explained in the Supplementary Information^41^. The SIBS pellets with a 25% styrene content were generously provided by InnFocus Inc., a Santen Company. To obtain the batch material of “magnetic SIBS”, a hot melt extrusion process with mixing was used (Fig. 2a). We ensured a homogeneous dispersion of the particles in the SIBS by thoroughly mixing in a mini twin-screw extruder heated at 150 °C at 100 rpm for 10 min. Microscopic imaging demonstrated that the obtained magnetic SIBS extruded pellets show a uniform dispersion of the particles in the SIBS matrix (see Figs. 2a and S1). The mold used in the hot embossing machine to give the magnetic SIBS a micropencil shape was fabricated using a femtosecond laser machining process (Fig. 2c). The design of the mold was prepared using dedicated Alphacam software, where the laser scanning path (tool-path) to be fed to the FEMTOprinter f200 aHead (FEMTOprint SA, Switzerland) for exposing the fused silica glass was also generated. The mold was fabricated on a 75 × 25 × 2-mm fused silica glass slide. The pulse energy and repetition rate used were 230 nJ and 1000 kHz, respectively. The laser was focused with a Thorlabs 20x microscope objective with a numerical aperture (NA) of 0.4. When the machining program was finished, the glass slide was immersed in a concentrated solution of 45% potassium hydroxide (KOH, Sigma‒Aldrich) diluted in water to remove the exposed material. Finally, the mold was rinsed thoroughly with acetone and DI water to remove all debris. To facilitate the release (demolding) of the magnetic micropencil plugs after the hot embossing step, the femtosecond laser-machined glass mold was first coated with a superhydrophobic layer of fluorosilane (Trichloro(1H,1H,2H,2H-perfluorooctyl)silane, Sigma‒Aldrich). To improve the adhesion of this coating, the mold underwent an oxygen plasma treatment performed immediately before fluorosilane vapor deposition. After the silanization treatment, the mold was ready to be used in the hot embossing machine (Specac limited) together with magnetic SIBS pellets to fabricate the micropencil plugs. We used 150 °C to melt the magnetic SIBS and 5 tons of pressure to help the melted polymer flow into the cavities of the mold (Fig. 2b). The demolding took place after the hot embossing had cooled to below 80 °C. After the hot embossing step, the residual layer attached to the micropencils was removed by cutting it with a razor blade under microscopic view.

### Fabrication of the device with an integrated microvalve

The mold used to fabricate the top and bottom layers of the micropencil devices was fabricated using the same femtosecond laser machining process as previously described. A fluorosilane coating was also applied. The mold was then used in the hot embossing machine together with the SIBS pellets for the fabrication of the microchip layers using the same protocol applied for the micropencil plugs. After demolding the patterned SIBS film, the microchip layers were cut apart using a razor blade, and a biopsy punch was used to create inlet and outlet connection holes. To integrate the magnetic micropencil into the device, a special coating was needed to prevent the micropencil from bonding to the actuation chamber walls during the thermal bonding step (used to make a closed device from the top and bottom layers). For this, a polyvinyl alcohol (PVA, molecular weight = 9000–10,000 g mol^−1^, Sigma‒Aldrich) solution (PVA: DI water = 1:10) was prepared, in which the micropencil plug was dip coated. For the sacrificial PVA layer to solidify, the coated plug was placed on a hot plate at 90 °C for 5 min. Subsequently, the PVA-coated plug was inserted into the actuation chamber, and the bottom and top layers were aligned. To facilitate the alignment process and promote perfect alignment of the layers, the top layer was designed to have four alignment pins, which fit into the alignment holes of the bottom layer. Due to the stickiness of SIBS, ethanol was used between the layers to enable their movement while aligning. After ethanol evaporation, the device was thermally bonded on a hot plate at 90 °C for 10 min while applying pressure with a weight placed on top of the device. When the bonding was complete, the PVA was easily removed by flushing the bonded device with DI water.

### Micropencil plug and device characterization

The shape and morphology and some key dimensions of the micropencil plug and implant channels were observed and measured using a Keyence VHX-5000 digital microscope.

### In vitro microfluidic experiments

Microfluidic experiments involving actuating the magnetic micropencil with a moving external magnet were carried out to confirm the valving function. The pressure upstream of the implant was measured while the microvalve switched between open/closed states. The setup used for this experiment is illustrated in Fig. 4a and comprised (1) a syringe pump (Fusion 200, Chemyx Inc.), pumping DI water at a constant flow rate of 2.5 µL/min – equal to the typical rate of aqueous humor production in the eye; (2) a pressure transducer (Omega Engineering), connected to the syringe pump and to the inlet of the device, thus constantly measuring the pressure upstream of the device (in mmHg); and (3) a column of water connected to the outlet of the device, mimicking the pressure in the bleb when hypotony is most likely to occur (approximately 1.38 mmHg, or 1.88 cmH_2_O). The magnet, with a geometry of 10 × 10 × 10 mm^3^ and a remnant flux density of 1.3 T, was positioned underneath the device at a distance of 1.75 mm. In between the device and the magnet, a 750-µm-thick polycarbonate sheet and a 1-mm-thick glass slide were used to hold the device.

We carried out different microfluidic experiments to fully characterize valve operation. First, an ON/OFF experiment was performed to check the repeatability of the valve function. In this experiment, the pressure was measured for approximately 5 min with the valve open and 30 min with the valve closed. In total, four ON/OFF cycles were performed for each sample. To investigate whether fluid can still leak through the main outlet channel when the valve is closed, devices without the main outlet channel and without the microvalve integrated were also fabricated and tested. Second, to evaluate the stability of the pressure when the valve is in the closed state, we measured the pressure upstream of the device for a period of 12 h. Third, to investigate whether valve function would be affected by the patient’s movements, we measured the pressure for a period of 12 h with the device placed under rocking motion using a digital platform rocker shaker (VWR). The rocking, side-to-side (2D) motion was performed in the main direction of the micropencil movement, and a tilt of 15° and a speed of 8 rpm (which corresponds to 16 side-to-side movements/minute) were used. Except for the nonstatic experiment, all the aforementioned experiments were performed in three samples/devices of both the 50 × 50-µm and 40 × 40-µm bypass channel devices.

### Ex vivo experiments

Ex vivo experiments were performed on porcine eyes, for which the actual implants comprised of a drainage tube and an elliptical-shaped end plate had to be first fabricated. The fabrication of the plate followed the same procedure that was used for making the microfluidic devices, i.e., the bottom and top layers were fabricated by replica molding using hot embossing and a femtosecond laser-machined glass mold. A homemade punching device with the same elliptical shape as the plate was used to remove the residual layer that was attached to the top and bottom layers after the hot embossing step. A solvent bonding technique was used to attach the drainage tube to the plate. For this, 1.5 mm of a 12-mm-long SIBS tube (100-µm inner diameter, 350-µm outer diameter), kindly provided by InnFocus Inc., was first dip-coated in toluene (98%, VWR Chemicals) and then immediately introduced into the channel of the plate specifically designed for the tube to be connected. Afterward, the same thermal bonding technique was used as previously explained. Finally, the device was thoroughly flushed with DI water to remove the PVA coating on the micropencil and any existing impurities.

Five freshly enucleated porcine eyes, with a postmortem time of less than 4 h, were obtained from a local butcher. For the implantation of the devices into the eyes, first, a fornix-based conjunctival flap was created, after which a deep scleral pocket was formed. Through the scleral pocket, a needle tract was made with a 25-gauge needle into the anterior chamber. The drainage tube was then implanted through the needle tract. At ~180° from the implantation site, the eyes were cannulated with another 25-gauge needle through which a saline solution of sodium chloride (NaCl 0.9%, B Braun) was injected into the anterior chamber at a constant flow rate of 20 µL/min delivered by a syringe pump; this specific flow rate was necessary to maintain physiological ocular rigidity throughout the experiment. The needle was inserted carefully between the anterior plane of the iris and the posterior surface of the cornea. In between the pump and the needle, a pressure transducer was connected to measure the pressure inside the eye (IOP) in real time. For each eye, one ON/OFF cycle was performed, and the pressure was measured for approximately 10 min with the valve open and 1 h with the valve closed. For comparison, the same experiment was also performed in vitro on the fabricated implants, where the same flow rate was applied.

## Supplementary information

Supplementary Information - clean version

Supplementary Video S1
