# Supplementary material for: Magnetically actuated glaucoma drainage device for regulating intraocular pressure after implantation
Source: Microsyst Nanoeng. 2023 Jul 20;9:92. doi: 10.1038/s41378-023-00561-9 (PMC10356933; doi:10.1038/s41378-023-00561-9)
Supplement: Supplementary file 1 — Supplementary Information - clean version [file 41378_2023_561_MOESM1_ESM.docx]

**Supplementary Information for**

Magnetically actuated glaucoma drainage device for regulating intraocular pressure after implantation

Inês C.F. Pereira^1,2^, Ralph J.S. van Mechelen^3^, Hans M. Wyss^1,2^, Leonard Pinchuk^4,5^, Henny J.M. Beckers^3^, Jaap M.J. den Toonder^1,2*^

^1^Microsystems, Department of Mechanical Engineering, Eindhoven University of Technology, 5600MB Eindhoven, The Netherlands

^2^Institute for Complex Molecular Systems (ICMS), Eindhoven University of Technology, 5600MB Eindhoven, The Netherlands

^3^University Eye Clinic Maastricht, Maastricht University Medical Centre+ (MUMC+), 6202AZ Maastricht, The Netherlands

^4^InnFocus, Inc., a Santen Company, Miami, Florida 33186, USA

^5^Ophthalmic Biophysics Center, Bascom Palmer Eye Institute, University of Miami Miller School of Medicine, Miami, Florida 33136, USA

^*^Correspondence to: Jaap M.J. den Toonder

**Email:** [j.m.j.d.toonder@tue.nl](mailto:j.m.j.d.toonder@tue.nl)

**This file includes:**

Supplementary text

Figures S1 to S5

Table S1

Legend for Movie S1

SI References

**Supplementary Information Text**

**Cytotoxicity.** The *in vitro* cytotoxicity of the magnetic poly(styrene-block-isobutylene-block-styrene) (magnetic SIBS) extracts on the human MG-63 osteosarcoma cell line was evaluated according to the ISO 10993-5-2009 standard. The cells were maintained in Minimum Essential Alpha Medium supplemented with 10% Fetal Bovine Serum (FBS), 100 U/mL penicillin and 100 µg streptomycin (now referred to as culture medium). MG-63 cells were cultured and refreshed every 2–3 days. Once the cells reached approximately 80% confluence, they were passaged. One day before the exposure to the extracts, the cells were trypsinized and resuspended in culture medium to create a suspension with a concentration of $1.8\times{10}^{4}$ cells/mL. Thereafter, 100 µL of the cell suspension was seeded into a 96-well plate, giving a seeding density of $1.8\times{10}^{3}$ cells/well. The cells were incubated for 24 hours at 37 °C with 5% CO_2_. The medium was then replaced by different magnetic SIBS extracts. For the preparation of the extracts, first, a 700 µm-thick magnetic SIBS film was fabricated using hot embossing and thereafter cut to small rectangular pieces of 10 x 5 mm. The samples were sterilized by immersing in 70% ethanol for 20 minutes. The prepared samples were rinsed in phosphate-buffered saline (PBS) and transferred to culture medium and incubated for 72 hours at 37 °C. The surface area to volume ratio was 3 cm^2^/mL for all samples (6 cm^2^/mL, if both top and bottom surfaces of the films are considered), in accordance with the ISO 10993-12-2012 standard. Three technical replicates were used for each sample. Dilutions were prepared to achieve 50%, 25% and 10% extracts. Undiluted, i.e., 100% extracts were also used. After 24h of incubation with the extracts, cell metabolic activity was evaluated using a resazurin (PrestoBlue) assay. The treatment medium was aspired and replaced with 90µL of culture medium and 10µL of PrestoBlue reagent. After 30 minutes of incubation, fluorescence at 530/590 nm (excitation/emission) was measured. Cytotoxicity of the extracts was depicted as a percentage of metabolic activity of the control, i.e., as relative metabolic activity (RMA) calculated as follows

| $RMA = 100 \times\frac{F_{\mathrm{sample}} - F_{\mathrm{blank}}}{F_{\mathrm{control}} - F_{\mathrm{blank}}}$ , | (1) |
| --- | --- |

where $F_{\mathrm{sample}}$ stands for the average fluorescence of resorufin produced by the affected cells (i.e., cells incubated with extracts), $F_{\mathrm{blank}}$ is the average fluorescence measured in the blank solution (no cells), and $F_{\mathrm{control}}$ stands for the average fluorescence of resorufin produced by of the unaffected cells (i.e., negative control cells). Extracts causing the fluorescent signal to decrease below 70% of the activity of the negative control were considered cytotoxic, as described in the standard ISO 10993-5. This experiment confirmed that the magnetic SIBS is non-cytotoxic at all the extract concentrations tested (**Fig. S2**).

**Femtosecond laser machining process and laser affected zone.** Although we designed the micropencil glass mold to yield micropencils with a diameter of 350 µm, following fabrication their diameter was 356 ± 1 µm, as can be seen in **Fig. 2d** in the main text. This minor deviation from the desired diameter can be explained by the size of the elliptical-shaped laser affected zone during the femtosecond laser exposure, which, in fused silica glass and when using a 20x objective, is 3 µm-wide and 24 µm-long ^1^. This will inevitably enlarge the diameter of the cavity in the glass mold exposed to the KOH by 3 µm, thus resulting in micropencil plugs with a larger diameter. Furthermore, after long exposure to KOH etching, the un-exposed parts of the glass also start to be etched. This may explain why the diameter of the plugs was increased even further. The length of the plugs varied quite significantly, with an average length of 988.3 ± 10 µm. However, these length differences were expected, since the residual layer attached to the cylindrical (bottom) portion of the plugs was manually removed with a razor blade after hot embossing, making it therefore very difficult to guarantee the same length across all the fabricated plugs.

For the channels in the devices, **Fig. S3e** shows that the height appears to have been less affected by the laser affected zone and/or KOH etching than the width. The reason for the height of the channels to be very similar to the initially designed one is that, in the glass mold, not only the top surface of the channel features is machined, but also the bottom surface, all around the channels. Machining the top surface reduces the height of the channels by approximately 12 µm, due to the size of the elliptical-shaped laser affected zone. In contrast, machining around the channel features, on the bottom surface of the mold, leads to a 12 µm-increase in the height of the channels. As the machining is done on both surfaces, the resultant change in the height from the originally designed one is almost negligible. On the other hand, machining both side walls of the channels will decrease their width by at least 3 µm due to the width of the laser affected zone, which can be aggravated by the KOH inadvertently etching the non-machined glass. This explains the smaller width obtained in all channels.

**Magnetic force calculation.** The translational force acting on the magnetic micropencil plug is calculated by first computing the magnetic field produced by the permanent magnet, with a geometry of 10x10x10 mm^3^ and a remnant flux density of 1.3 T, using the commercially available software COMSOL Multiphysics. The magnetic flux density and its variation (gradient) is extracted from the simulated model to then calculate the net driving force acting on the plug, which is given by

| $\vec{F}_{\mathrm{mag}}=(\vec{m}\cdot\nabla)\vec{B}=\rho V\left( \vec{M_{0}}\cdot\nabla\right)\vec{B}+\frac{V\chi}{\mu_{0}}(\vec{B}\cdot\nabla)\vec{B}$ , | (2) |
| --- | --- |

where $\rho$ is the density of the magnetic particles (7.86 $\text{g}/{\text{cm}^{\text{3}}}$), $V$ is the volume of magnetic particles in a micropencil plug ($\text{1.4×}\text{10}^{\text{-5}} \text{cm}^{\text{3}})$, $\chi$ is the magnetic susceptibility of the magnetic particles (here we take $\chi=0.4$ ^2^), $\mu_{0}$ is the permeability of vacuum, $\vec{M_{0}}$ is the initial magnetization of the plug and $\vec{B}$ is the applied magnetic field. $\vec{M_{0}}$ is neglected since it has a value very close to zero. As a result, the above equation becomes

| $\vec{F}_{\mathrm{mag}}=\frac{V\chi}{\mu_{0}}(\vec{B}\cdot\nabla)\vec{B}$ . | (3) |
| --- | --- |

From this equation, we conclude that the magnetic translational force applied to the micropencil plug is the combined effect of the magnitude of the magnetic field and the magnetic field gradient experienced by the plug. As the plug has a single degree of freedom (linear translation), we calculated the magnetic translational force along its direction of movement. At a vertical distance of 2.15 mm from the surface of the magnet and horizontal distance varying from 0–8mm with respect to the center axis of the magnet, we estimated that the magnetic force along the direction of the plug movement varies approximately from $\text{1.97×}\text{10}^{\text{-6}}$ N (magnitude of the magnetic field $\text{B}$ = 0.35 T, magnitude of the magnetic gradient $\text{∇}\text{B}$ = 45.56 T/m), when the center axis of the plug is perfectly aligned with the center axis of the magnet, to a maximum force of $\text{5.67×}\text{10}^{\text{-5}}$ N ($\text{B}$ = 0.27 T, $\text{∇}\text{B}$ = 88.77 T/m) when the magnet is at a distance of 5 mm from the plug (corner of the magnet is aligned with the central axis of the plug, as depicted in **Fig. 4b** in the main text). This force is obtained if we consider that the magnet is positioned with the north-pole facing up (vertical polarity).

**Hydrodynamic resistances calculations.** We theoretically determined the total hydrodynamic resistance of our micropencil devices ($r_{\mathrm{total}}$) by taking the sum of the hydrodynamic resistances of the inlet channel ($r_{\mathrm{inlet}}$), the parallel bypass and main outlet channels ($r_{\mathrm{bypass}}$, $r_{main outlet}$), and the combined outlet channel ($r_{combined outlet}$, where the bypass and main outlet channels come together, see **Fig. 3** and **Fig. S3**), as follows

| $r_{\mathrm{total}} = r_{\mathrm{inlet}}+ \frac{r_{\mathrm{bypass}} \times r_{main outlet}}{r_{\mathrm{bypass}} + r_{main outlet}} + r_{combined outlet}$ . | (4) |
| --- | --- |

To calculate the hydrodynamic resistance of each channel ($r_{\mathrm{channel}}$), the following formula was applied

| $r_{\mathrm{channel}} = \frac{12 \mu L}{1 - 0.63(h/w)} \frac{1}{h^{3}w}$ , | (5) |
| --- | --- |

where $\mu\left[ \text{Pa s} \right]$ is the dynamic viscosity of the aqueous humor, $L$ is the length of the channel, and $h$ and $w$ represent, respectively, the average height and width of the channel measured beforehand (see **Fig. S3e**). The $r_{\mathrm{total}}$ was determined for the devices with the valve both in the open and in the closed states, as well as for the devices containing only the bypass channel and no main outlet channel. Since $r_{\mathrm{total}}$ is the difference between the applied outlet pressure and measured upstream pressure, divided by the applied flow rate, then

| $r_{\mathrm{total}} = \frac{IOP - P_{\mathrm{bleb}}}{Q}=r_{\mathrm{inlet}} + \frac{r_{\mathrm{bypass}} \times r_{main outlet}}{r_{\mathrm{bypass}} + r_{main outlet}} + r_{combined outlet}$ , | (6) |
| --- | --- |

where $IOP$ is the pressure measured experimentally, $P_{\mathrm{bleb}}$ is the applied outlet pressure, i.e., the pressure in the bleb when hypotony is most likely to occur (approximately 1.38 mmHg), and $Q$ is the flow rate applied (2.5 µL/min). From equation (6), the $r_{main outlet}$ for the experiments with the valve closed, which effectively equals the hydrodynamic resistance provided by the valve ($r_{\mathrm{valve}}$), was calculated as follows

| $r_{main outlet} = \frac{- r_{\mathrm{bypass}} \times(\frac{IOP - P_{\mathrm{bleb}}}{Q} - r_{\mathrm{inlet}} - r_{combined outlet})}{\left( \frac{IOP - P_{\mathrm{bleb}}}{Q} - r_{\mathrm{inlet}} - r_{combined outlet} \right) - r_{\mathrm{bypass}}}$ . | (7) |
| --- | --- |

The results from these calculations are shown in **Table S1**. Confirming the consistency of the calculations, we find that the estimated hydrodynamic resistance provided by the valve ($r_{\mathrm{valve}}$) is almost identical for the two types of devices. This indicates that the valve behaves in a similar manner and is independent of the bypass channel dimensions. When the valve is open, the total hydrodynamic resistance ($r_{\mathrm{total}}$) is very similar between devices, which is expected since the hydrodynamic resistance is then dominated by the open main outlet channel, which is identical for both devices. On the other hand, and as anticipated, the total hydrodynamic resistance of the 40x40 µm device when the valve is closed is approximately two times higher than that of the 50x50 µm device (4.53 as compared to 2.22 $\text{mmHg}/{\text{µL min}^{\text{-1}}}$). When the valve is in the open state, the hydrodynamic resistance of our devices is lower than that theoretically calculated for the PRESERFLO MicroShunt of 1.81 $\text{mmHg}/{\text{µL min}^{\text{-1}}}$ ^3^. This means that our device might possibly produce a more significant IOP-lowering effect compared to that of the PRESERFLO MicroShunt, which has already been proven successful in reducing IOP in glaucoma patients’ eyes ^4,5^.

Using the calculated hydrodynamic resistance provided by the valve, we estimated the pressure that would be experimentally measured if different inflow rates were used. As mentioned earlier, the *in vitro* experiments were performed using an average inflow rate of 2.5 µL/min. However, the aqueous humor production rate varies slightly between the waking and sleeping hours. In a healthy person, aqueous humor is produced at approximately 3 μL/min in the morning, 2.5 μL/min in the afternoon, and 1.5 μL/min during the night ^6^. Based on our calculations, if the valve is in the closed state and the lower value of aqueous flow rate (1.5 μL/min) is considered, the IOP would be 4.70 mmHg for the 50x50 µm device and 8.18 mmHg for the 40x40 µm device. On the other hand, if the higher flow rate (3 μL/min) is considered and the valve is closed, the IOP would be 8.03 mmHg and 14.98 mmHg for the 50x50 µm and 40x40 µm devices, respectively. These values do not significantly deviate from the healthy IOP range of 5–15 mmHg, meaning that if the aqueous humor production rate varies slightly with the circadian rhythm, both types of devices with the valve in the closed state would still provide acceptable IOP values.

**Critical Particle Volume Concentration (CPVC) principle.** The amount of magnetic particles, carbonyl iron powder (CIP, 99.5%, average diameter of 5 µm, Sigma-Aldrich), to be mixed with SIBS was pre-determined following the Critical Particle Volume Concentration (CPVC) principle ^7^. The critical volume concentration of particles in an elastomeric matrix is the concentration at which the particles are in physical contact with each other and the voids between them are completely filled with the elastomer. At concentrations lower than the CPVC, the particles are separated by more elastomer, while for higher concentrations than the CPVC, the amount of elastomer is not sufficient to completely surround the particles. In the latter case, the mechanical properties, such as stress at break, may be deteriorated, and the composite material stiffens due to the increase in filler content. The CPVC for a magnetic powder can be calculated from its apparent/bulk density. When iron particles are stored in a container, there will be a large amount of air in between the particles. This will mean that the “apparent density” is much less than the density of the solid iron particles. If the air is replaced totally by the elastomeric matrix, the result is the elastomer filled with a critical amount of iron. For the CPVC calculation, the following formula can be used

| ${CPVC}_{\mathrm{CIP}} = 100 \times\frac{\rho_{\mathrm{apparent}}}{\rho_{\mathrm{iron}}}$ , | (8) |
| --- | --- |

where $\rho_{\mathrm{apparent}}$ is the apparent density of the CIP and $\rho_{\mathrm{iron}}$ is equal to 7.86, which is the density of iron ($\text{g}/{\text{cm}^{\text{3}}}$). $\rho_{\mathrm{apparent}}$ varies between 3–4 $\text{g}/{\text{cm}^{\text{3}}}$, and we considered the value of 3.50 $\text{g}/{\text{cm}^{\text{3}}}$ in our calculations. According to the formula, the CPVC of CIP in the SIBS matrix is 44.53 vol%. We have decided to fabricate our magnetic SIBS with a weight ratio of 1:2, where the concentration of particles in the SIBS matrix is approximately half (≈ 20 vol%) of the CPVC, in order to preserve the mechanical properties of the composite.

Supplementary Information Figures and Tables


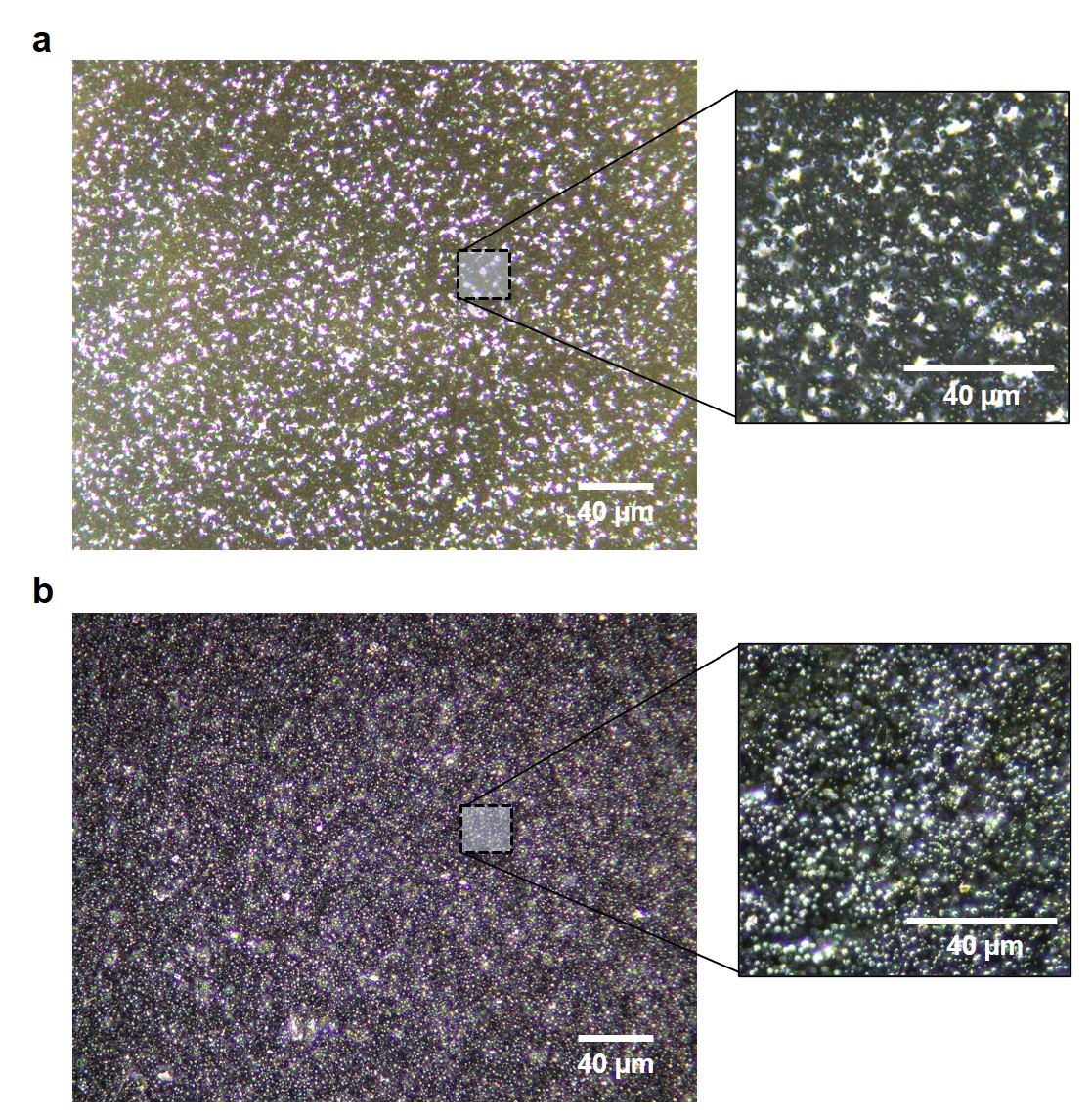


**Fig. S1.** Microscopic images showing the dispersion of the magnetic particles in the SIBS matrix, captured using varying magnifications and using both transmission (**a**) and reflection (**b**) modes for imaging the samples. A 100 µm-thin magnetic SIBS film fabricated upfront using hot embossing was used to take these images.


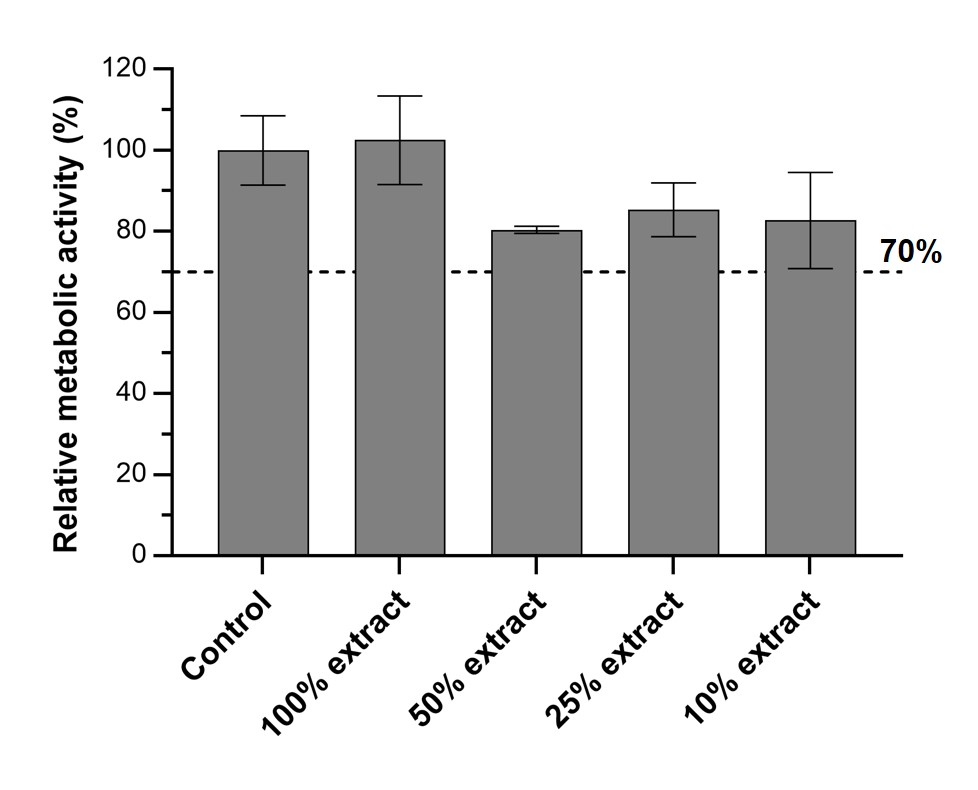


**Fig. S2.** Relative metabolic activity of the human MG-63 osteosarcoma cells (resazurin assay) after 24 h incubation with 100%, 50%, 25% and 10% extracts of magnetic SIBS. The data represents the mean ± standard deviation (SD) (*n* = 3). Sole extraction medium served as a control (unaffected cells which metabolic activity was taken as 100%). The dashed line indicates the normative limit of 70% metabolic activity of the control (ISO 10993-5-2009).


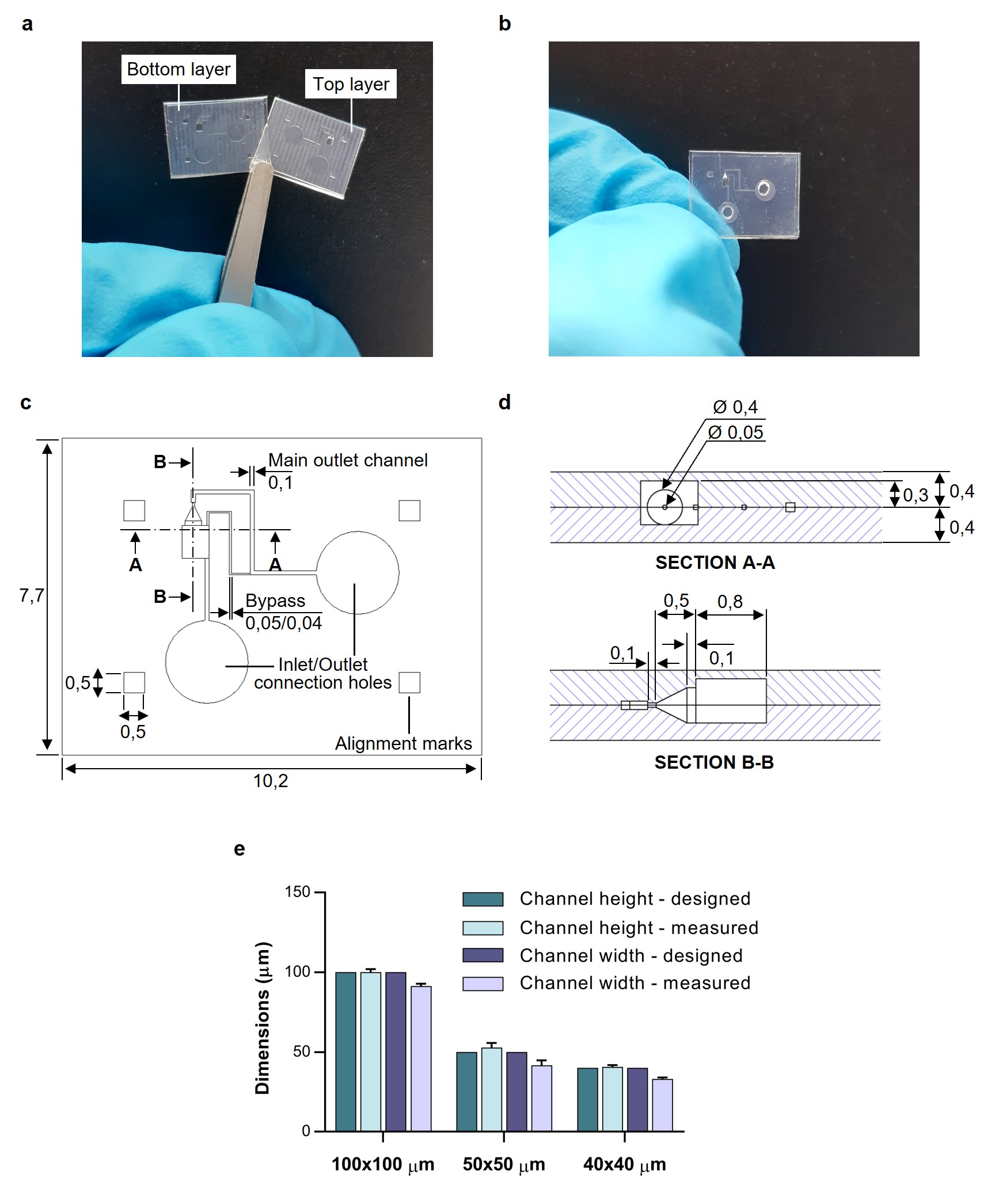


**Fig. S3.** Micropencil device design and relevant dimensions. (**a**) Replica-molded top and bottom layers, and (**b**) the bonded micropencil device used for the *in vitro* experiments. (**c**) Schematic top view of the channel layout and relevant dimensions in mm. (**d**) Cross-sectional views of the device showing the actuation chamber shape and key dimensions in mm. (**e**) Graph indicating the differences between the measured channel dimensions and the initially designed ones. The data represents the mean ± SD (*n* = 3).


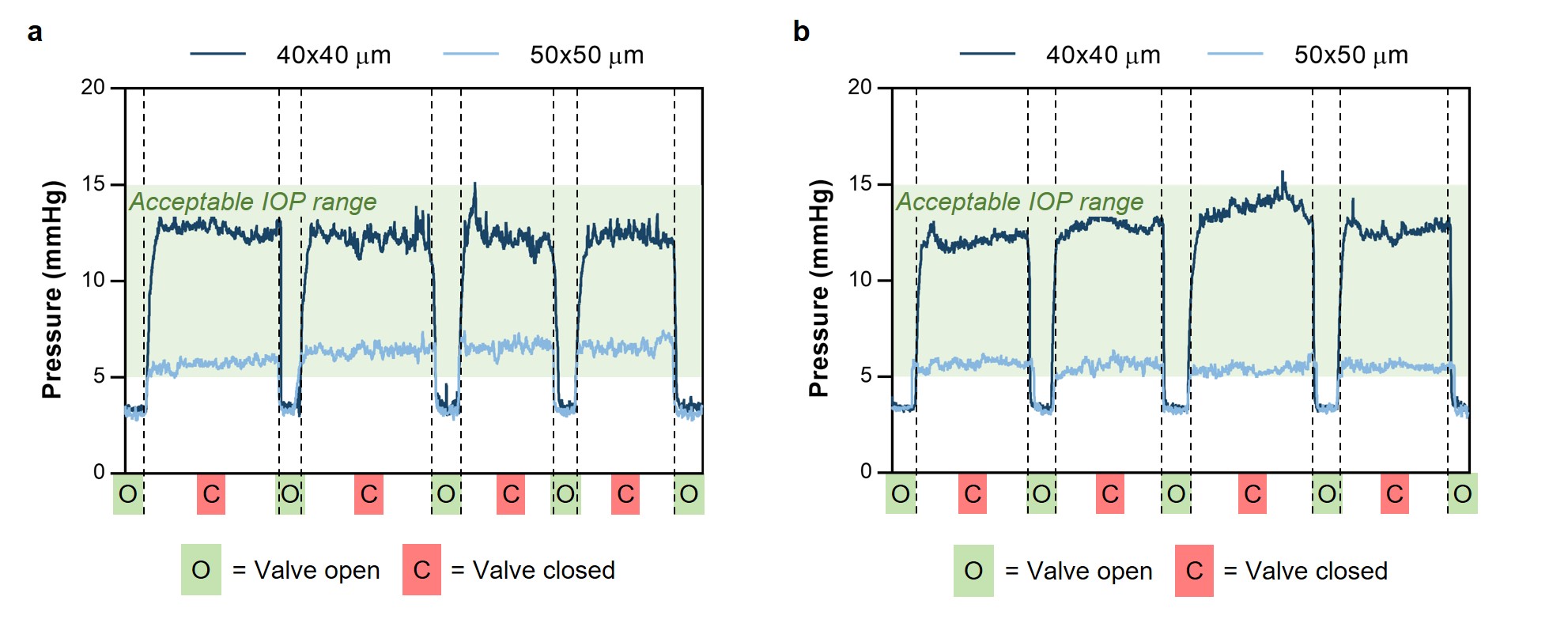


**Fig. S4.** *In vitro* measurement of the pressure variation upstream the 40x40 µm and 50x50 µm micropencil devices – (**a**) Samples 2 and (**b**) Samples 3 – as a result of the valve operation. The shaded green areas represent an acceptable IOP range of 5–15 mmHg.


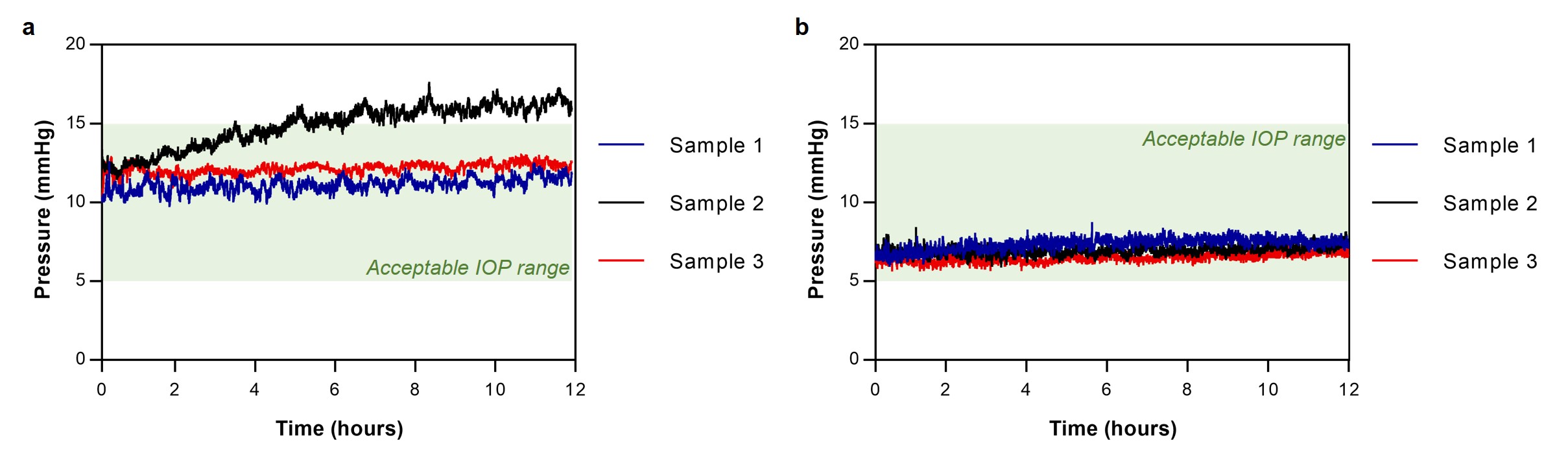


**Fig. S5.** Overview of the pressures measured *in vitro* upstream the (**a**) 40x40 µm and (**b**) 50x50 µm micropencil devices for period of 12 hours with the valve in the closed state, and under static conditions (i.e., the devices did not move over the course of the experiment). Each line represents one of the samples tested. The shaded green areas represent an acceptable IOP range of 5–15 mmHg.

**Table S1.** Hydrodynamic resistance of microfluidic devices used in the *in vitro* experiments.

| Bypass channel size (height x width) | Condition | $\boldsymbol{r}_{\mathbf{total}}$ | $\boldsymbol{r}_{\mathbf{valve}}$ | Units |
| --- | --- | --- | --- | --- |
| 50x50 µm | Only bypass channel | 2.50 | 15.18 | $\text{mmHg}/{\text{µL min}^{\text{-1}}}$ |
|  | Valve open | 0.45 |  |  |
|  | Valve closed | 2.22 |  |  |
| 40x40 µm | Only bypass channel | 6.24 | 14.89 |  |
|  | Valve open | 0.46 |  |  |
|  | Valve closed | 4.53 |  |  |

**Movie S1 (separate file).** Demonstration of the microvalve switching between open and closed states by moving the micropencil plug using an external magnet.

**SI References**

1. Rajesh, S. & Bellouard, Y. Towards fast femtosecond laser micromachining of fused silica: The effect of deposited energy. *Opt. Express* **18**, 21490–21497 (2010).

2. Shevkoplyas, S. S., Siegel, A. C., Westervelt, R. M., Prentiss, M. G. & Whitesides, G. M. The force acting on a superparamagnetic bead due to an applied magnetic field. *Lab Chip* **7**, 1294–1302 (2007).

3. Barberá, M. I. *et al.* Evaluation of the Ultrastructural and In Vitro Flow Properties of the PRESERFLO MicroShunt. *Transl. Vis. Sci. Technol.* **10**, 26 (2021).

4. Batlle, J. F., Corona, A. & Albuquerque, R. Long-term Results of the PRESERFLO MicroShunt in Patients with Primary Open-angle Glaucoma from a Single-center Nonrandomized Study. *J. Glaucoma* **30**, 281–286 (2021).

5. Scheres, L. M. J. *et al.* XEN® Gel Stent compared to PRESERFLO^TM^ MicroShunt implantation for primary open-angle glaucoma: two-year results. *Acta Ophthalmol.* **99**, e433–e440 (2021).

6. Goel, M., Picciani, R. G., Lee, R. K. & Bhattacharya, S. K. Aqueous Humor Dynamics: A Review. *Open Ophthalmol. J.* **4**, 52–59 (2010).

7. Lokander, M. & Stenberg, B. Performance of isotropic magnetorheological rubber materials. *Polym. Test.* **22**, 245–251 (2003).
